# Maternal Health-Related Quality of Life and Its Predicting Factors in the Postpartum Period in Iran

**DOI:** 10.1155/2016/8542147

**Published:** 2016-02-28

**Authors:** Nazanin Rezaei, Arman Azadi, Razieh Zargousi, Zinab Sadoughi, Zahra Tavalaee, Maryam Rezayati

**Affiliations:** ^1^Psychosocial Injuries Research Center, Ilam University of Medical Sciences, Ilam 6939177143, Iran; ^2^Department of Nursing, Faculty of Nursing and Midwifery, Ilam University of Medical Sciences, Ilam 6939177143, Iran; ^3^Family and Population Health Department, Ilam University of Medical Sciences, Ilam 6939177143, Iran; ^4^Department of Midwifery, Faculty of Nursing and Midwifery, Ilam University of Medical Sciences, Ilam 6939177143, Iran

## Abstract

*Background/Purpose*. Postpartum period is accompanied by many physical, emotional, and social changes in women's health. The aim of this study was to examine the mothers' quality of life in postpartum period. In addition, it also sought to recognize the variables that predict their quality of life.* Methods*. This cross-sectional, descriptive study was undertaken among 380 women in 10 urban health centers in Ilam province in west of Iran. They were selected using proportional random sampling method. The SF-36 questionnaire was used to identify women's health-related quality of life (HRQoL). Data were analyzed using SPSS version 15.* Results*. Women who were employed, aged less than 30 years, had college degree, have no history of disease in pregnancy, and had given birth more than 3 months ago had higher quality of life scores. Independent predictors for lower physical HRQoL were being with history of disease in pregnancy; being with high school diploma or lower education; and giving birth less than 3 months ago. Also, independent predictor for lower mental HRQoL was being housewife.* Conclusion*. According to study findings, greater attention must be paid to providing postpartum healthcare for housewife and less educated women as well as those with history of disease in pregnancy.

## 1. Introduction

While health care professionals are crucially involved in prenatal care, postpartum period is a neglected aspect of women's health care [1, 2]. Although a great deal of research has been conducted in recent decades to explore various aspects of postnatal care, more remains to be done [3, 4]. Not surprisingly, the content and relevance of postpartum health care have been criticized as inadequate to meet the health needs of mothers. For example, a routine 6-week postpartum follow-up for the mother in GB has conventionally focused on vaginal examinations or family planning more than their physical and psychological health [5]. Postpartum care also terminates while mothers are still struggling to adapt with new roles and changes in the family environment [6].

Postpartum-related morbidities have been reported in studies from several countries [1, 7, 8]. Fatigue or tiredness is the most prevalent postpartum physical health problems experienced by more than half of mothers [4]. Fatigue has been found to be positively associated with postpartum depression and breastfeeding problems [8]. Pain in various parts of bodies is another frequent symptom [4]. Incontinence, hemorrhoids, constipation, sleeping disorders, and a variety of emotional changes such as depressive symptoms are examples of other conditions that affect mothers' physical and psychological health [4, 9–11]. These health problems not only influence mothers' health but also affect their infants' well-being [12]. However, these problems are often dismissed by health professionals as “only to be expected” [5]. Similarly, such problems might also be neglected by both family members and health professional due to the concentration on child care during the early months [13]. So, inadequate postpartum surveillances may adversely affect mothers as well as infants quality of life.

Quality of life (QoL) is a term that is used in many studies, mainly in the health care field [14]. QoL is a broad multidimensional and dynamic concept that affects performance of the individual in physical, psychological, social, and spiritual aspects of life [15]. World Health Organization (WHO) describes QoL as “the individual's perception of their life in the context of the culture and value systems in which they live and in relation to their goals, expectations, standards, and concerns” [16]. QoL has become an area of increasing importance to the area of maternal and child health [3]. Women's perception of their health-related quality of life is an essential measure of the quality and effectiveness of maternal and child health interventions [5].

To the best of our knowledge, there are inadequate studies that examined postpartum HRQoL of Iranian women. Most of current studies focused on QoL associated with certain types of delivery [1, 6] and the other influencing and predicting factors that may affect mothers QoL are not well investigated. Most of research regarding the postpartum QoL has been conducted only in the United States and other developed countries. Additionally, as noted before QoL is a concept affected by culture and social systems [15, 17]. So, the results of studies in western countries are often not applicable in eastern contexts. Accordingly, the aim of this study was to examine the mothers QoL in postpartum period and its relationship with sociomaternal factors. In addition, it sought to recognize the variables that predict QoL of Iranian women.

## 2. Material and Methods

### 2.1. Study Design

This cross-sectional, descriptive study was conducted in 10 urban health centers in Ilam province in west of Iran.

### 2.2. Study Population

The study population included all women who met the following criteria: (a) less than 6 months passing since the delivery; (b) aging ≥18 years; (c) willing to participate in the study; and (d) living in Ilam in previous year. Women with obstetric/neonatal complications related to the current birth, postpartum depression, and any disabling chronic illness were excluded from the study.

### 2.3. Selection of Study Subjects

Sampling was performed using randomized cluster sampling. First, Ilam city five geographical zones (central, north, south, east, and west) were sampled by randomly selecting two urban health centers per zone. Each zone was considered to be a cluster. Since the number of households covered by each urban health center was different, proportional random sampling was used to determine the sample size of the ten centers. After reviewing family health records women who met criteria for the study were identified and eligible cases were selected randomly and invited to participate in the study. The sample size (*n* = 375) was calculated based on the power of 0.8 and *α* of 0.05, and *σ* = 22 [18]. Considering sample attrition, a sample of 400 eligible women were invited to participate in the study. Overall, 380 subjects accepted to be enrolled (response rate = 95%) and filled the questionnaires.

### 2.4. Study Instrument

The instrument for data collection is composed of two main parts. The first part was a checklist of socioindividual status of women and was completed based on patients' self-report or their medical records. Demographic data included age, the number of living children, the type of delivery, occupation, disease history, educational level, and history of divorce. The second part was health-related quality of life (HRQoL) questionnaire. This multidimensional instrument was developed by John Ware in 1992. The scale covers eight dimensions of HRQoL: physical function (PF, 10 items), role limitations due to physical problems (RP, 4 items), bodily pain (BP, 2 items), general health (GH, 5 items), vitality (VT, 4 items), social function (SF, 2 items), role limitation due to emotional problems (RE, 3 items), and perceived mental health (MH, 5 items). The scale scores within each domain were ranging from 0 (worst imaginable health state) to 100 (best imaginable health state). The reliability and validity of Iranian version of this questionnaire were assessed by Montazeri et al. (2005) using internal consistency and known-groups comparison and convergent validity [19]. The Cronbach alpha coefficients in eight aspects of the scale were ranging from 0.77 to 0.90 [19]. The scales face and content validity were assessed and verified by the expert panel composed of ten faculty members affiliated to Ilam and Tabriz Universities of Medical Sciences in Iran. The final version of the questionnaire was tested for reliability in a pilot study involving 25 postpartum women. Cronbach alpha coefficient values in eight aspects of the scale were ranging from 0.74 to 0.88.

### 2.5. Ethical Issues and Data Collection

Before the data collection, the study proposal was approved by the regional ethics committee of Ilam University of Medical Sciences (EC/93/H/259). Next, researchers were referring to the urban health centers from March 2014 to June 2015. After presenting basic information, the study subjects were asked to participate in a private interview for data collection. Interviews were performed by a female interviewer in the subjects preferred place. All women who participated in the study gave informed consent.

### 2.6. Statistical Analysis

Data were analyzed using SPSS version 15. Descriptive statistics such as the frequency, percentage, mean, and standard deviation were used to describe the demographic/health-related quality of life of women. The *t*-test was used to perform bivariate analysis to check the association between the demographic/obstetric characteristics of postpartum women and their HRQoL. A logistic regression model was used to determine predictors of postpartum HRQoL of study subjects. The input variables to the model (assumed predictors of HRQoL) included age (<30 versus >30 years), level of education (>12 versus <12 years), employment (housewife versus employed), disease in pregnancy, breastfeeding, and time elapsed since birth (<3 versus >3 months). Statistical significance level was set at *p* < 0.05.

## 3. Results

Mean (SD) age of subjects was 29.81 (5.5). Most of women were housewife (86.6) and about half of them (44.7%) had a college degree. More than half of them (66.3%) reported income more than expense. About half of women were primiparous (50.5) and the type of delivery was mainly cesarean (61.3%). Cesarean delivery was higher among those with a college degree, primiparous, and had a higher household income. However, there were no significant differences between postpartum HRQoL and type of delivery. Majority (81.1%) of women reported receiving 6–8 prenatal visits and 89.2% had breastfeeding.  Table 1 shows some sociodemographic characteristics of subjects.

The participants' scores in eight aspects of HRQoL according to their demographics and obstetric characteristics are shown in Table 2. As shown in Table 2, the mean scores of 3 dimensions of HRQoL (VT, SF, and BP) were significantly higher among women aged less than 30 years. Women with a college degree had higher scores in MH and PF than women with high school diploma or lower degree. Also, employed mothers had significantly higher scores in VT, MH, RE, and PF subscales. Women with the history of disease in pregnancy had lower HRQoL on all dimensions (except for RP and SF) of HRQoL. No significant differences were observed between the mean scores of seven dimensions (except for RE) of HRQoL among women who breastfed and those who did not. The mean scores of HRQoL in BF and RP dimensions were significantly higher among women who had given birth more than 3 months ago. Women with 6–8 prenatal visits reported higher scores in GH and VT subscales than those with irregular prenatal visits.

The input variables recognized by regression analysis as statistically significant predictors of HRQoL of postpartum women are listed in Table 3. Independent predictors for lower physical HRQoL (mean score of PF + BP + RP + GH subscales) were being with history of disease in pregnancy (OR: 10.43; 95% CI: 2.32–46.92); being with high school diploma or lower education (OR: 1.60; 95% CI: 1.04–2.45); and giving birth less than 3 months ago (OR: 1.57; 95% CI: 1.006–2.45). Also, independent predictor for lower mental HRQoL (mean score of VT + MH + SE + RE subscales) was being housewife (OR: 2.12; 95% CI: 1.07–4.17). Neither breastfeeding nor educational status was significant predictors of postpartum mental QoL.

## 4. Discussion

The aim this study was to examine the mothers' QoL in postpartum period and its relationship with sociomaternal factors. According to extensive literature review, this is one of the first studies which assessed women's QoL in postpartum period and its predictor factors in Iranian context. The study findings showed that these women had moderate scores in most of HRQoL subscales. Some other national and international studies have reported similar findings [7, 17]. Therefore, the finding of this study highlighted the importance of existing evidence regarding the insufficiency of content and relevance of postpartum health care for mothers.

The study findings showed that there were no differences between postpartum HRQoL of women after normal vaginal delivery and cesarean section. This is incongruence with the findings reported in some previous related studies in Iran and other countries [1, 12, 20–22]. For example, the findings of Torkan et al. (2009) study showed that in general women with normal vaginal delivery had better quality of life than those with cesarean section [1]. However, in Mousavi et al. study (2013) there was no difference between QoL scores after a cesarean section and vaginal delivery [23]. Such discrepancies in literature may be due the fact that postpartum quality of life is influenced by many factors other than type of delivery, such as socioeconomic background and mother/child-related factors. Also, using different scales for measuring QoL by researchers make it difficult to compare the findings.

According to the study findings, women who had regular prenatal visit reported higher QoL scores which is consistent with the findings of other studies [10, 22]. Incongruent with the findings of previous studies we could not find any association between women's QoL and their experience of having more than 2 children [3, 10]. In our study women aged younger than 30 years had better QoL in three subscales of SF-36. However, in a study conducted among Brazilian women there was no difference between QoL scores of postpartum women in different age groups [10]. We do not know why this happened but perhaps one might argue that younger women are mostly primiparous and receiving more attention by both family members and health professionals. Women with a college degree had higher scores in mental health and physical function subscales which is similar to Bahrami et al. (2014) findings in Dezful, Iran.

Although household income is an important factor for better QoL [17], in our study there was no difference between women QoL and their monthly incomes. This may be due to their tending to not disclose their real financial situation. Consistent with other studies, history of disease in pregnancy affects mothers QoL on 7 dimensions of SF-36 questionnaire, especially physically related subscales [10, 21, 22]. The association between disease in pregnancy and lower QoL score in the present study was further supported by the results of the regression analysis.

As another finding of the study, employed women had better score in half of HRQoL subscales than housewife women. However, in de Oliveira et al. (2015) study there was no difference between postpartum women's QoL and their employment. In another study which examined health of employed women at 5 weeks postpartum most of them reported moderate QoL [22]. In current study breastfeeding was reported by most of the study participants. But (except for role emotional subscale) there was no difference between women's QoL and breastfeeding. McGovern et al. (2006) also reported no association between breastfeeding and mental QoL of postpartum women. In that study breastfeeding was also associated with significantly more physical symptoms, such as breast discomfort, increased fatigue, and excessive sweating [22]. However, Zubaran and Foresti (2011) found a significant relationship between the breastfeeding efficacy and QoL of postpartum women in Brazil [24]. The findings of a systematic review also showed a negative association among breastfeeding and postpartum depression [25].

Consistent with the findings of Bahrami et al. (2014) who found gradual improvement in QoL of postpartum women with increasing time since birth, our findings also showed that women who gave birth more than 3 months ago had higher QoL scores in both bodily pain and role physical subscales. This is also supported by Wang et al. (2013) and Dalfrà et al. (2012) who reported improvement of postpartum physical functioning over time [26, 27]. However, in Li et al. (2014) study the effect of time since birth on HRQoL of postpartum women was not significant [21].

Numerous variables have been suggested by other studies as predictors of HRQoL in postpartum women, such as race, marital status, maternity leave, baby's gender, parity, better preconception health, increased perceived control, and vaginal (versus cesarean section) delivery [4, 6, 10, 22, 27]. However, we used several simple predictors as the input variables based on results of related studies assumed as specific predictors of HRQoL in postpartum women. Using these variables has the benefit of making our prediction model simple and easy to use. Our logistic regression model showed that the physical HRQoL scores in postpartum women was a function of history of disease in pregnancy, education, and time elapsed since birth. In our study women with history of disease in pregnancy reported much higher bodily pain than others. However, the type of delivery in most of mothers with history of disease in pregnancy was cesarean section; therefore, higher bodily pain was an expected outcome. McGovern et al. (2006) found that physical health of postpartum women was associated with vaginal delivery and more time since birth. However, in our study the type of delivery was not predictor of HRQoL in postpartum period.

Factors suggested by other studies as predictors of better postpartum mental health included social support, breastfeeding, better preconception health, no sign of prenatal mood problems, and having a baby girl [10, 22]. However, in our study the only significant predictor of mental HRQoL of these postpartum women was their employment status. Consistent with our findings, in Wang et al. (2013) study, women who were employed perceived having better general health than those who were unemployed [27].

The study finding has implications for health professionals who are working in obstetrics and other health centers. Enhancing the participation of postpartum mothers in the nature of their care may lead to better provision of health care and improvement in their QoL. Current conventionally postpartum care, such as routine examination of vagina, blood pressure, body temperature, and family planning consultation, should be substituted with a wide range of related health and social need assessment including physical and mental health as well as considering social and cultural issues. Predictors of HRQoL among postpartum women in our study are consistent with the findings of previous studies. So, our findings should be considered for designing effective health promotion strategies that allow women to maintain optimal health for themselves and their infants. In our study employed women and those with higher education had better QoL than other women. These findings may be due to the fact that employed and educated women have higher health knowledge and also better access to health care facilities. So, greater attention must be paid to providing postpartum healthcare for housewife and less educated women as well as those with history of disease in pregnancy.

Some limitations of our study should be acknowledged. First, finding relationships is difficult to evaluate in cross-sectional studies, so longitudinal studies are needed to precisely examine which factors may affect postpartum women's health. Second, based on our sampling method study subjects were recruited from a variety of geographical zone in Ilam city. However, this sample does not represent variation of all the country population. In relation to future research, replicating such studies in other Iranian provinces as well as Middle East countries is required. As aforementioned many postpartum women especially in developing countries do not receive appropriate care. Accordingly, there is need for evidence on the effects of existing models of postpartum care on women health and quality of life.

## Figures and Tables

**Table 1:** Sociodemographic and maternal characteristics of subjects.

Characteristics	*N* (%)
Age (years)	
15–25	74 (19.5%)
25–35	234 (61.6%)
>35	72 (18.9%)
Education (years)	
<12	210 (55.3%)
>12	170 (44.7%)
Job	
Housewife	329 (86.6%)
Employed	51 (13.4%)
Number of deliveries	
1-2	338 (88.9%)
>2	42 (11.1%)
Disease in pregnancy	
Yes	362 (95.3%)
No	18 (4.7%)
Income	
Fair	252 (66.3%)
Poor	128 (33.7%)
Type of delivery	
NVD	147 (38.7%)
CS	233 (61.3%)
Breastfeeding	
Yes	339 (89.2%)
No	41 (10.8%)
Time since birth (months)	
<3	128 (33.7%)
>3	252 (66.3%)
Time since marriage (years)	
1–3	159 (41.8%)
>3	222 (58.2%)

NVD: normal vaginal delivery; CS: cesarean section.

**Table 2:** The participants' scores in eight aspects of HRQoL according to their demographics and obstetric characteristics.

Characteristics	Number	Subscales of SF-36							
		General health	Vitality	Mental health	Role emotional	Social function	Bodily pain	Role physical	Physical function
Age (years)									
<30	216	50.88 (14.2)	62.13 (19.1)	69.41 (18.8)	62.50 (38.8)	70.54 (21.5)	66.03 (19.8)	60.30 (32.7)	67.01 (26.2)
>30	164	49.63 (14.2)	58.14 (19.1)	66.61 (19.1)	59.35 (40.6)	66.16 (21.9)	61.53 (21.8)	55.49 (37.7)	68.41 (25.2)
*p*		0.396	0.044	0.142	0.438	0.048	0.036	0.198	0.60
Education (year)									
>12	170	51.29 (14.5)	62.60 (19.3)	71.38 (18.5)	64.71 (40.2)	68.53 (22.2)	63.89 (21.1)	58.09 (37.5)	71.56 (23.1)
<12	210	49.57 (13.8)	59.07 (19.0)	65.67 (18.0)	58.25 (38.1)	68.75 (21.5)	64.26 (20.6)	58.33 (34.9)	64.43 (27.3)
*p*		0.238	0.131	0.003	0.110	0.922	0.866	0.948	0.007
Employment									
Employed	51	52.25 (13.9)	65.78 (17.6)	73.80 (14.8)	72.54 (36.9)	70.34 (20.9)	66.90 (21.2)	64.21 (37.5)	74.90 (23.4)
Housewife	329	50.04 (14.1)	59.57 (19.3)	67.33 (18.7)	59.37 (39.2)	68.39 (21.9)	63.66 (20.7)	57.29 (35.7)	66.49 (25.2)
*p*		0.30	0.031	0.019	0.025	0.552	0.301	0.202	0.03
Disease in pregnancy									
No	366	50.68 (14.1)	60.94 (18.8)	68.70 (18.2)	62.02 (39.1)	69.06 (21.6)	64.90 (20.3)	58.74 (36.1)	68.32 (25.5)
Yes	14	41.43 (13.9)	46.43 (23.8)	55.14 (19.6)	38.09 (34.2)	58.03 (25.3)	43.14 (23.6)	44.64 (34.2)	49.28 (26.9)
*p*		0.016	0.005	0.007	0.025	0.063	0.000	0.151	0.006
Breastfeeding									
Yes	339	50.24 (14.5)	60.20 (19.1)	67.69 (18.2)	59.30 (39.4)	68.36 (21.8)	63.53 (20.6)	57.15 (36.7)	66.89 (25.8)
No	41	51.23 (11.3)	62.20 (19.5)	72.40 (18.5)	76.40 (33.5)	71.04 (22.1)	68.78 (21.9)	67.07 (32.3)	73.66 (24.7)
*p*		0.675	0.528	0.123	0.008	0.459	0.127	0.096	0.112
Time since birth (months)									
>3	252	50.44 (14.0)	61.73 (18.6)	68.41 (18.2)	62.83 (38.7)	69.94 (21.9)	65.61 (20.7)	61.64 (35.0)	67.80 (26.0)
<3	128	50.16 (14.4)	57.81 (20.1)	67.78 (18.9)	58.81 (40.0)	66.11 (21.3)	61.00 (20.6)	51.95 (37.3)	67.20 (25.3)
*p*		0.855	0.061	0.752	0.238	0.106	0.038	0.015	0.833

Values are expressed as mean (SD).

**Table 3:** Logistic regression analysis of factors associated with physical^a^ and mental^b^ HRQoL scores of postpartum women in Iran.

Parameter	Crude OR (95% CI)	Adjusted OR (95% CI)	*p* value
History of disease in pregnancy	8.83 (2.00–38.99)	10.43 (2.32–46.92)	0.002
Education			
(Reference: <12 years)			
>12 years	1.54 (1.02–2.31)	1.60 (1.04–2.45)	0.03
Time since birth			
(Reference: >3 months)			
<3 months	1.43 (0.937–2.20)	1.57 (1.006–2.45)	0.047
Employment			
(Reference: housewife)			
Employed	2.20 (1.17–4.13)	2.12 (1.07–4.17)	0.03

CI: confidence interval; OR: odds ratio.

^a^Physical HRQoL referred to mean score of PF + BP + RP + GH. Independent predictors were history of disease in pregnancy, education, and time elapsed since birth.

^b^Mental HRQoL referred to mean score of VT + MH + SE + RE. Independent predictor was employment.
